# Assessing the quality and readability of patient education materials on chemotherapy cardiotoxicity from artificial intelligence chatbots: An observational cross-sectional study

**DOI:** 10.1097/MD.0000000000042135

**Published:** 2025-04-11

**Authors:** Christoph A. Stephenson-Moe, Benjamin J. Behers, Rebecca M. Gibons, Brett M. Behers, Laura De Jesus Herrera, Djhemson Anneaud, Manuel A. Rosario, Caroline N. Wojtas, Samantha Bhambrah, Karen M. Hamad

**Affiliations:** a Florida State University College of Medicine, Tallahassee, FL; b Florida State University Internal Medicine Residency, Sarasota Memorial Hospital, Sarasota, FL; c University of South Florida College of Medicine, Tampa, FL.

**Keywords:** artificial intelligence, chatbots, chemotherapy cardiotoxicity, patient education materials

## Abstract

Artificial intelligence (AI) and the introduction of Large Language Model (LLM) chatbots have become a common source of patient inquiry in healthcare. The quality and readability of AI-generated patient education materials (PEM) is the subject of many studies across multiple medical topics. Most demonstrate poor readability and acceptable quality. However, an area yet to be investigated is chemotherapy-induced cardiotoxicity. This study seeks to assess the quality and readability of chatbot created PEM relative to chemotherapy-induced cardiotoxicity. We conducted an observational cross-sectional study in August 2024 by asking 10 questions to 4 chatbots: ChatGPT, Microsoft Copilot (Copilot), Google Gemini (Gemini), and Meta AI (Meta). The generated material was assessed for readability using 7 tools: Flesch Reading Ease Score (FRES), Flesch-Kincaid Grade Level (FKGL), Gunning Fog Index (GFI), Coleman-Liau Index (CLI), Simple Measure of Gobbledygook (SMOG) Index, Automated Readability Index (ARI), and FORCAST Grade Level. Quality was assessed using modified versions of 2 validated tools: the Patient Education Materials Assessment Tool (PEMAT), which outputs a 0% to 100% score, and DISCERN, a 1 (unsatisfactory) to 5 (highly satisfactory) scoring system. Descriptive statistics were used to evaluate performance and compare chatbots amongst each other. Mean reading grade level (RGL) across all chatbots was 13.7. Calculated RGLs for ChatGPT, Copilot, Gemini and Meta were 14.2, 14.0, 12.5, 14.2, respectively. Mean DISCERN scores across the chatbots was 4.2. DISCERN scores for ChatGPT, Copilot, Gemini, and Meta were 4.2, 4.3, 4.2, and 3.9, respectively. Median PEMAT scores for understandability and actionability were 91.7% and 75%, respectively. Understandability and actionability scores for ChatGPT, Copilot, Gemini, and Meta were 100% and 75%, 91.7% and 75%, 90.9% and 75%, and 91.7% and 50%, respectively. AI chatbots produce high quality PEM with poor readability. We do not discourage using chatbots to create PEM but recommend cautioning patients about their readability concerns. AI chatbots are not an alternative to a healthcare provider. Furthermore, there is no consensus on which chatbots create the highest quality PEM. Future studies are needed to assess the effectiveness of AI chatbots in providing PEM to patients and how the capabilities of AI chatbots are changing over time.

## 1. Introduction

Artificial intelligence (AI) has become a valuable tool in healthcare due to its ability to learn and recognize patterns from large datasets.^[1]^ This function has allowed it to assume roles ranging from compiling vast amounts of patient data to aiding radiologists and pathologists in reading studies to even serving as a source of inquiry for patients. Its use in serving as a source of inquiry for patients has expanded of late with the introduction of large language model (LLM) chatbots. Chatbots have increased in prevalence following the widespread adoption of OpenAI’s ChatGPT, with other technology companies releasing iterations of their own. They are popular amongst users due to their ability to respond to specific inquiries with evidence-based answers, fast output, and ease of use.^[2]^

Given their abilities and accessibility, it seems inevitable that chatbots will continue to play an increasing role in patient education. Numerous studies have assessed the quality and readability of patient education materials produced by various AI chatbots. The general consensus of these studies is that the information is written with too much complexity and the quality is highly variable.^[3]^ However, given the constantly updating and evolving nature of chatbots, along with the wide range of medical topics that have yet to be investigated, continued studies on the quality and readability of these materials are important.

One topic that has yet to be investigated is chemotherapy-induced cardiotoxicity. Chemotherapy cardiotoxicity is associated with the use of trastuzumab and doxorubicin, both of which are agents for breast cancer, with the latter also being used for leukemias and lymphomas, as well as other metastatic cancers.^[4]^ Aside from skin cancer, breast cancer is the most commonly occurring cancer in women, with an estimated 316,950 new cases in the United States (US) in 2025.^[5]^ Cardiotoxicity from doxorubicin occurs in a dose-dependent manner with risks ranging from 5% to 26% depending on dose, while trastuzumab is associated with a risk ranging between 0% to 3%.^[6]^ The increasing prevalence of cancer in the population makes this topic especially important. Furthermore, a 2018 study investigating the prevalence of internet usage among cancer patients found that 59.1% of respondents used the internet to search for cancer related information, which was second only to physicians as a source of information at 60.3 %.^[7]^ Given the prevalence of the internet as a source of information and the importance of chemotherapy induced cardiotoxicity, this study seeks to assess the quality and readability of AI chatbot-generated patient education materials on chemotherapy cardiotoxicity.

## 2. Methods

We Google searched “chemotherapy cardiotoxicity” in August 2024 and used the first result, Cleveland Clinic, to identify questions surrounding chemotherapy-induced cardiotoxicity.^[8]^ The Cleveland Clinic is a world-renowned hospital and academic medical center who treated over 3 million patients from all over the world in 2023, with a main campus is in Cleveland, Ohio, USA. It is staffed with experts in all fields of medicine, serving as a source of innovation and education.^[9]^ In August 2024, we asked each chatbot the 10 questions posed on the Cleveland Clinic page in consecutive order (Table 1). Four chatbots were used in this study: ChatGPT Version 3.5 (OpenAI, San Francisco, CA), Microsoft Copilot (Microsoft, Redmond, WA), Google Gemini 1.0 Pro (Google DeepMind, London, England, UK), and Meta AI Version 24.0 (Meta, New York, NY). These chatbots were chosen because of their broad popularity and use, as well as presence in other similar studies of this kind. Questions and their responses were copied into a Microsoft Word (version 15.30) document, creating patient education materials for each chatbot. Readability assessments were performed using an online readability tool, while quality was assessed using the Patient Education Materials Assessment Tool (PEMAT) and DISCERN questionnaire.^[10–12]^

**Table 1:** The 10 questions asked to each chatbot.

Questions
1. What is chemotherapy cardiotoxicity?
2. Who does chemotherapy cardiotoxicity affect?
3. How does chemotherapy cardiotoxicity affect my body?
4. What are the symptoms of chemotherapy cardiotoxicity?
5. What causes chemotherapy cardiotoxicity?
6. How is chemotherapy cardiotoxicity diagnosed?
7. How is chemotherapy cardiotoxicity treated?
8. How can I reduce my risk of chemotherapy cardiotoxicity?
9. Can chemotherapy cardiotoxicity be reversed?
10. When should I see my healthcare provider?

Questions sourced from Cleveland Clinic website: https://my.clevelandclinic.org/health/diseases/16858-chemotherapy--the-heart-cardiotoxicity.

### 2.1. Readability

Readability was assessed across 7 commonly used measures: Flesch Reading Ease Score (FRES), Flesch-Kincaid Grade Level (FKGL), Gunning Fog Index (GFI), Coleman-Liau Index (CLI), Simple Measure of Gobbledygook (SMOG) Index, Automated Readability Index (ARI), and FORCAST Grade Level.^[13–21]^ FRES uses the length of words and sentences in a document to provide a score between 1 and 100, with a target score of 60 or greater.^[13,14]^ Lower scores using the FRES tool indicate material that is more difficult to read. The other 6 measures indicate the necessary number of years completed of United States (US) schooling to understand a text.^[13–21]^ The formulas used to calculate each measure can be found in Table 2. Means with standard deviations (SD) were calculated for the 7 readability scores across all chatbots and for the average reading grade level across the 6 tools for each chatbot.

**Table 2:** Formulae for computing readability scores for chatbots

Tool	Formula
Flesch Reading Ease score (FRES)^[13,14]^	FRES = 206.835–1.015(TW/TS)–84.6(TSy/TW) •Scores: 90-100 (Grade 5), 80-90 (Grade 6), 70-80 (Grade 7), 60-70 (Grades 8-9), 50-60 (Grades 10-12), 30-50 (College), 0-30 (College Graduate)
Flesch-Kincaid Grade Level (FKGL)^[13,14]^	FKGL = 0.39(TW/TS) + 11.8(TSy/TW)–15.59
Gunning Fog Index (GFI)^[13,15]^	GFI = 0.4 x [(TW/TS) + 100(CW/TW)]
Coleman-Liau Index (CLI)^[13,16]^	CLI = (0.0588 × L)–(0.296 × S)–15.8
Simple Measure of Gobbledygook (SMOG) Index^[13,17]^	SMOG = 3 + √(Polysyllabic Count)
Automated Readability Index (ARI)^[13,18,19]^	ARI = 4.71(TC/TW) + 0.5(TW/TS)–21.43
FORCAST Grade Level^[13,21]^	FORCAST = 20–(N/10)

Table 2. Higher FRES scores indicate material is at a lower reading grade level, interpretation of scores can be seen in above table. The scores of the other 6 tools indicate a direct estimation of the materials reading grade level. Reading grade level is the estimated number of years of United States schooling to understand the text. Higher reading grade levels indicate material that is relatively more difficult to read.

TW = Number of Total Words in the Document, TS = Number of Total Sentences in the Document, TSy = Number of Total Syllables in the Document, CW = Number of Complex Words in the Document (Complex Words are words with 3 or more syllables), L = Average Number of Letters per 100 Words in the Document, S = Average Number of Sentences Per 100 Words in the Document, Polysyllabic Count = Number of Words in the Document with 3 + Syllables, TC = Number of Total Characters in the Document, N = Number of Single-Syllable Words in a 150-Word Sample of the Document.

### 2.2. Quality

PEMAT and DISCERN are widely used, validated tools to assess the quality of patient education materials.^[11,12]^ Each of the 4 AI-generated documents were independently analyzed using these instruments by 2 of the contributing authors (C.S.M. and B.J.B.). Disagreements were resolved through discussion, with K.M.H. available to serve as an independent mediator for any that could not be resolved.

### 2.3. PEMAT-P

PEMAT evaluates patient education materials across 26 Items: 19 Items for Understandability and 7 Items for Actionability.^[11]^ We used a modified PEMAT for printed materials (PEMAT-P) that excludes items pertaining to audio information (Items 13–14). We further modified the PEMAT-P to exclude items on the use of visual aids (Items 15–19 and 25–26), as AI-generated responses do not provide figures or other visual aids. This resulted in 12 Items for Understandability (Items 1–12) and 5 Items for Actionability (Items 20–24). The modified PEMAT-P instrument used in this study can be found in Table 3. Each item received a score of 1 if the criteria were met and 0 if not. Items received a score of “N/A” if the criteria were not applicable to the material (e.g., an item assessing the quality of an infographic when no infographic is present). Each chatbot document was given a separate Understandability and Actionability score. Scores for each section were calculated by total score received divided by total number of applicable items, multiplied by 100%. Final scores were assigned as “pass” or “fail” using a ≥ 70% cutoff per PEMAT guidelines.

**Table 3:** The modified PEMAT-P instrument used in this study. Item numbers are labeled how they appear on the original PEMAT instrument

Item #	Item	Response options
1	The material makes its purpose completely evident.	Disagree = 0, Agree = 1
2	The material does not contain material that distracts from its purpose.	Disagree = 0, Agree = 1
3	The material uses everyday common language.	Disagree = 0, Agree = 1
4	Medical terms are used only to familiarize the audience with the terms. When used, medical terms are defined.	Disagree = 0, Agree = 1
5	The material uses the active voice.	Disagree = 0, Agree = 1
6	Numbers appearing in the material are clear and easy to understand.	Disagree = 0, Agree = 1 No numbers = N/A
7	The material does not expect the user to perform calculations.	Disagree = 0, Agree = 1
8	The material breaks or “chunks” information into short sections	Disagree = 0, Agree = 1 Very short material = N/A
9	The material’s sections have informative headers.	Disagree = 0, Agree = 1 Very short material = N/A
10	The material presents information in a logical sequence.	Disagree = 0, Agree = 1
11	The material provides a summary.	Disagree = 0, Agree = 1 Very short material = N/A
12	The material uses visual cues (e.g., arrows, boxes, bullets, bold, larger font, highlighting) to draw attention to key points.	Disagree = 0, Agree = 1 Video = N/A
20	The material clearly identifies at least 1 action the user can take.	Disagree = 0, Agree = 1
21	The material addresses the user directly when describing actions	Disagree = 0, Agree = 1
22	The material breaks down any action into manageable, explicit steps.	Disagree = 0, Agree = 1
23	The material provides a tangible tool (e.g., menu planners, checklists) whenever it could help the user take action.	Disagree = 0, Agree = 1
24	The material provides simple instructions or examples of how to perform calculations.	Disagree = 0, Agree = 1, No calculations = NA

### 2.4. DISCERN

DISCERN is a validated tool to assess the quality of written healthcare information concerning treatment choices.^[12]^ The tool contains 16 questions: questions 1 to 8 assess reliability, questions 9 to 15 assess quality of information, and question 16 assesses overall performance.^[12]^ Each question is scored between 1 (material does not satisfy item criteria and is unsatisfactory) and 5 (material completely satisfies item criteria and is highly satisfactory). Scores of 3 represent partially satisfied criteria and is satisfactory, while scores of 2 and 4 represent subjective intermediates. We excluded questions pertaining to sources of the information provided (questions 4, 5, and 7), as chatbots do not routinely provide references. Additionally, we modified the DISCERN so that question 16 was a calculated average of the previous 12 question scores, as opposed to a subjective 1–5 score. Our modified DISCERN contained 13 questions: questions 1–5 (correlating to DISCERN items 1–3,6, and 8) investigated reliability, questions 6–12 (correlating to DISCERN items 9–15) investigated quality, and question 13 (correlating to DISCERN item 16) determined overall performance. A list of the questions asked can be found in Table 4. Scores were reported as averages for the reliability and quality sections, as well as the overall average across all questions. Means with standard deviations were calculated for overall scores across the 4 chatbots for all 3 sections.

**Table 4:** The modified DISCERN instrument questions with the original DISCERN number listed in parenthesis

Modified DISCERN Instrument Questions
1. Are the aims clear? (1)
2. Does it achieve its aims? (2)
3. Is it relevant? (3)
4. Is it balanced and unbiased? (6)
5. Does it refer to areas of uncertainty? (8)
6. Does it describe how each treatment works? (9)
7. Does it describe the benefits of each treatment? (10)
8. Does it describe the risks of each treatment? (11)
9. Does it describe what would happen if no treatment is used? (12)
10. Does it describe how the treatment choices affect overall quality of life? (13)
11. Is it clear that there may be more than one possible treatment choice? (14)
12. Does it provide support for shared decision-making? (15)
13. Overall quality? (16)

### 2.5. Data analysis

We used descriptive statistics, such as frequencies and means with standard deviations to evaluate the quality and readability of chatbots, as well as compare the 4 chatbots amongst each other.

## 3. Results

### 3.1. Readability

**Figure 1.**
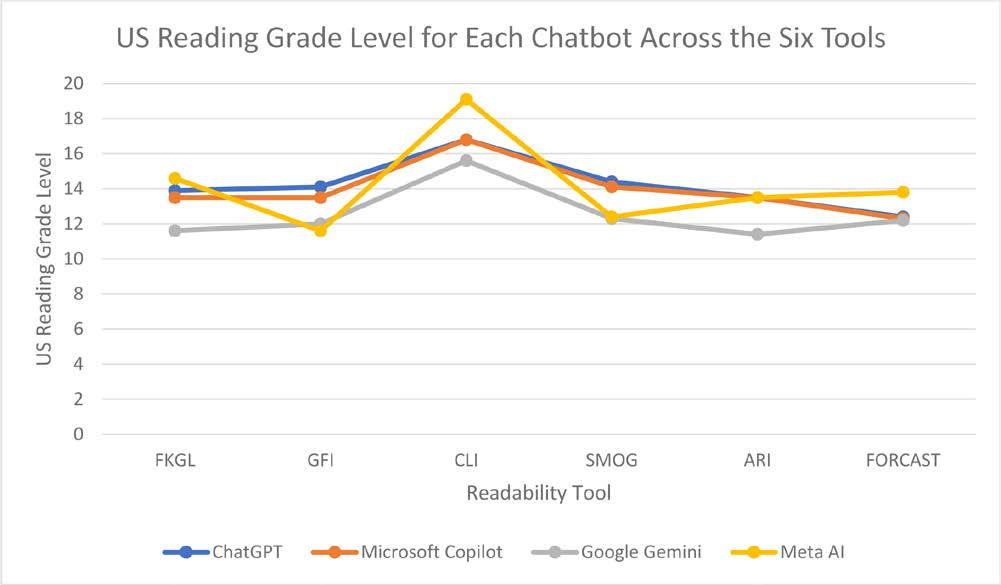
Line chart, representing readability scores (y axis) of chatbot created material across 6 different tools (x axis) was created using Microsoft Excel, FKGL = Flesh-Kincaid Grade Level, GFI = Gunning Fog Index, CLI = Coleman-Liau Index, SMOG = Simple Measure of Gobbledygook, ARI = Automated Readability Index.

FRES scores for ChatGPT, Microsoft Copilot, Google Gemini, and Meta AI were 21.5, 23.3, 32, and 4.3, respectively, with an overall mean of 20.3, and standard deviation (SD) of 11.6, which equates to US college graduate level (FRES of 0–30). Overall mean reading grade level of the 4 chatbots across all 6 tools was 13.7. The mean reading grade levels across the 6 readability tools ─ FKGL, GFI, CLI, SMOG, ARI, and FORCAST ─ for ChatGPT, Microsoft Copilot, Google Gemini, and Meta AI were 14.2 (SD = 1.5), 14.0 (SD = 1.5), 12.5 (SD = 1.6), and 14.2 (SD = 2.6), respectively. The readability scores for each chatbot across each of the 7 measures can be found in Table 5. A graphical representation of the reading grade levels for each chatbot across the 6 measures can be found in Figure 1.

**Table 5:** Readability scores of the patient education materials produced by the 4 chatbots across the 7 measures

Readability tool	ChatGPT	Microsoft Copilot	Google Gemini	Meta AI	Mean (SD)
FRES	21.5	23.3	32.0	4.3	20.3 (11.6)
FKGL	13.9	13.5	11.6	14.6	13.4 (1.3)
GFI	14.1	13.5	12	11.6	12.8 (1.2)
CLI	16.8	16.8	15.6	19.1	17.1 (1.5)
SMOG	14.4	14.1	12.3	12.4	13.3 (1.1)
ARI	13.5	13.5	11.4	13.5	13.0 (1.1)
FORCAST	12.4	12.3	12.2	13.8	12.7 (0.8)
Mean Grade Level (SD)	14.2 (1.5)	14.0 (1.5)	12.5 (1.6)	14.2 (2.6)	

Table 5. The column Mean Grade level represents the mean of the 6 readability scores listed above (FKGL, GFI, CLI, SMOG, ARI, and FORCAST) and does not include the FRES score as part of its calculation.

Lower FRES scores indicate that material is more difficult to read. Scores between 0 and 30 represent a reading grade level equivalent to that of a college graduate, scores between 30 and 50 indicate a reading level of a college student. Scores from the other 6 readability tools (FKGL, GFI, CLI, SMOG, ARI, and FORCAST) represent the estimated number of years of United States schooling to understand the text, with a score of above twelve indicating the reading level of a college student.

### 3.2. PEMAT

Across the 12 PEMAT Understandability Items, median chatbot score was 91.7%. ChatGPT had an Understandability score of 100%, while Microsoft Copilot and Meta AI received Understandability scores of 91.7% for meeting criteria on 11 of the 12 Items. Only 11 Items were applicable to Google Gemini, and it met criteria on 10 of these for an Understandability score of 90.9%. Item 6 was not applicable to Google Gemini due to its responses not having numbers. Overall, final Understandability scores for all chatbots exceeded 70%, indicating a passing PEMAT score.

Across the 5 PEMAT Actionability Items, the median chatbot score was 75%. ChatGPT, Microsoft Copilot, and Google Gemini received Actionability scores of 75% for meeting criteria on 3 of the 4 applicable Items. Meta AI received an Actionability score of 50% for meeting criteria on 2 of the 4 applicable Items. Item 24 was not applicable to any of the 4 chatbots due to the lack of calculations in these patient education materials on chemotherapy cardiotoxicity. Overall, final Actionability scores for ChatGPT, Microsoft Copilot, and Google Gemini exceeded 70%, indicating a passing PEMAT score, while Meta AI did not pass with its score of 50%. The PEMAT scores for the chatbots can be found in Table 6.

**Table 6:** PEMAT scores for the 4 chatbots

	ChatGPT	Microsoft Copilot	Google Gemini	Meta AI
1. The material makes its purpose completely evident.	1	1	1	1
2. the material does not contain material that distracts from its purpose.	1	1	1	1
3. The material uses everyday common language.	1	1	1	1
4. Medical terms are used only to familiarize the audience with terms. When used, medical terms are defined.	1	1	1	1
5. The material uses the active voice.	1	1	1	1
6. Numbers appearing in the material are clear and easy to understand.	1	1	N/A	1
7. The material does not expect the user to perform calculations.	1	1	1	1
8. The material breaks or “chunks” information into short sections.	1	1	1	1
9. The material’s section have informative headings.	1	1	1	1
10. The material presents information in a logical sequence.	1	1	1	1
11. The material provides a summary.	1	0	0	0
12. The material uses visual cues (e.g. arrows, boxes, bullets, bold, large font, highlighting) to draw attention to key points.	1	1	1	1
20. The material clearly identifies at least one action the user can take.	1	1	1	1
21. The material address the user directly when describing actions.	1	1	1	0
22. The material breaks down any action into manageable, explicit steps.	1	1	1	1
23. The material provides a tangible tool (e.g., menu planners, checklists) whenever it could help the user take action.	0	0	0	0
24. The material provides simple instructions or examples of how to perform calculations.	N/A	N/A	N/A	N/A
Understandability (Items 1–12)	100% (12/12)	91.7% (11/12)	90.9% (10/11)	91.7% (11/12)
Actionability (Items 20–24)	75% (3/4)	75% (3/4)	75% (3/4)	50% (2/4)

Passing score is ≥ 70%.

### 3.3. DISCERN

Across all 4 chatbots, mean DISCERN scores for reliability, quality, and overall were 4.7, 3.7, and 4.2, respectively. DISCERN scores across the 3 sections for each chatbot can be found in Table 7.

**Table 7:** DISCERN scores for the 4 chatbots

DISCERN section	ChatGPT	Microsoft Copilot	Google Gemini	Meta AI	Mean (SD)
Section 1: Reliability	4.6	4.8	4.8	4.6	4.7 (0.12)
Section 2: Quality	3.9	3.9	3.7	3.4	3.7 (0.24)
Overall Score	4.2	4.3	4.2	3.9	4.2 (0.17)

### 3.4. Reliability: questions 1–5

All chatbots had highly satisfactory scores for questions 1 through 4. Microsoft Copilot and Google Gemini had above satisfactory scores, while ChatGPT and Meta AI had satisfactory scores for question 5.

### 3.5. Quality: questions 6–12

All chatbots had highly satisfactory scores on questions 11 and 12, satisfactory scores on question 9, and unsatisfactory scores on question 8. ChatGPT and Microsoft Copilot had highly satisfactory scores on question 6 and above satisfactory scores on questions 7 and 10. Google Gemini had a highly satisfactory score on question 10, an above satisfactory score on question 6, and a satisfactory score on question 7. Meta AI had an above satisfactory score on questions 7 and 10 and a below satisfactory score on question 6.

### 3.6. Overall

**Figure 2.**
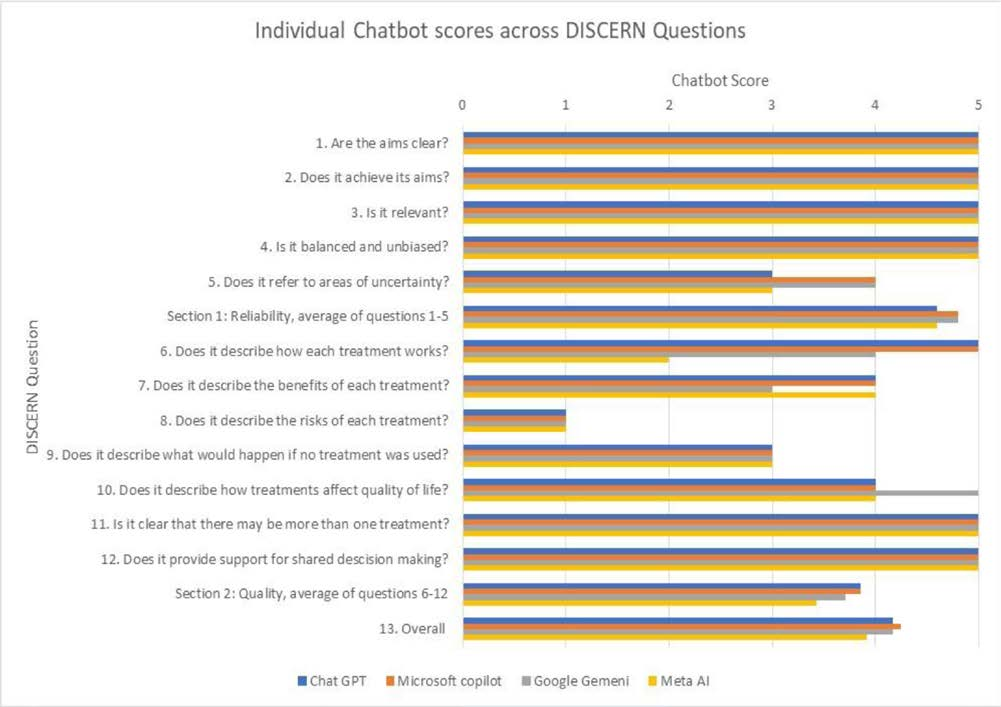
Bar chart created in Microsoft Excel, representing individual chatbot DISCERN scores (x axis) to each question (y axis) from our modified DISCERN tool.

Overall DISCERN scores for ChatGPT, Microsoft Copilot, Google Gemini, and Meta AI were 4.2, 4.3, 4.2, and 3.9, respectively. Individual chatbot scores across the DISCERN questions can be found in Figure 2.

## 4. Discussion

This study sought to evaluate the quality and readability of AI-generated responses to frequently asked questions about chemotherapy-induced cardiotoxicity. Our results indicate that AI chatbots produce high-quality patient education materials on this topic, although at a complex reading level. Readability was found to be at a college to college-graduate reading level, depending on the readability tool used. On the other hand, our PEMAT results indicated high quality material in terms of understandability and actionability, while DISCERN results indicated high reliability and quality. Overall, Google Gemini provided the most readable education materials on chemotherapy cardiotoxicity, as it had the lowest mean reading grade level. Microsoft Copilot had the highest DISCERN scores across all 3 measures (reliability, quality, overall), while ChatGPT received the highest PEMAT scores (Understandability, Actionability, Overall).

### 4.1. Readability

Across the 4 chatbots, mean reading grade level was 13.7, corresponding with college-level reading material. Mean FRES was 20.3, indicating college graduate reading level, well below the target goal of 60. Amongst individual chatbots, Google Gemini had the lowest mean reading grade level across the scoring tools with 12.5, while ChatGPT and Meta AI had the highest at 14.2. These are all well above the complexity recommended by the National Institutes of Health (NIH) and the American Medical Association (AMA), who hold that health education material should not exceed a 6th grade reading level.^[22]^ Prior studies analyzing patient education materials produced by AI chatbots have achieved similar results to ours in terms of readability. One study assessing the quality and readability of information related to benign prostatic hyperplasia surgery from 4 chatbots (ChatGPT, Bard, Bing AI, and Doximity GPT) yielded a mean FKGL of 12.1.^[23]^ Another study assessing 100 cancer-related queries posed to 4 AI chatbots (ChatGPT, Perplexity, Chatsonic, and BingAI) found a median reading grade level of 12.7.^[24]^ A third study assessing readability of patient education materials on cardiac catheterization produced by 4 chatbots (ChatGPT, Microsoft Copilot, Google Gemini, and Meta AI) yielded a mean FRES of 40.2, corresponding to college reading level.^[3]^ In line with our study, the patient education materials produced in these studies were all at the college reading level, representing a significant limitation to the use of AI chatbots for patient education materials in the general public.

It is important to note that web-based patient education materials can also suffer from low readability scores. One study assessing the quality and readability of 57 websites on nasopharyngeal carcinoma found they had a mean reading grade level of 14.3 and mean FRES of 53.2, corresponding with college and 10^th^ to 12^th^ grade levels, respectively.^[25]^ Similarly, another study assessing the quality and readability of patient education materials on esophageal cancer across 108 websites found they had a median FRES of 48.25, again corresponding to college reading level.^[26]^ These findings suggest that readability problems are not unique to AI chatbots. Given the similar reading levels between web-based and chatbot patient education materials, the readability issues could be due to the challenge of putting complex medical information into terms understandable by the general public. For instance, in the United States, roughly 90% of adults over the age of 65 have a high school degree, while just under 50% have a college degree.^[27]^ Despite the educational achievements of elderly adults, who are most likely to require patient education material on cancer-related topics, the NIH and AMA still recommend all material be at a 6th grade reading level, as previously mentioned. The chatbots we investigated were all readily available without subscription payment and intended for public use. Although we did not directly compare the readability of AI-generated and web-page based material, our results suggest that these 4 chatbots may be capable of producing material with similar readability.

### 4.2. PEMAT and DISCERN

Despite the readability concerns, quality assessments using validated patient education material assessment tools suggest that all 4 chatbots produced high-quality materials. All 4 chatbots had passing PEMAT Understandability scores, while only Meta AI had a failing Actionability score. For PEMAT Understandability, Meta AI received a perfect score, while the other 3 chatbots did not meet criteria for item 11, indicating that they failed to provide a summary of the material. All 4 chatbots did not meet criteria for item 23, indicating that none of their responses provided a tangible tool to help the user take action. Additionally, Meta AI did not meet criteria for Item 20, indicating the material did not clearly identify at least 1 action the use can take. Actionability scores were consistently lower than understandability scores. Mean DISCERN scores for reliability, quality, and overall were 4.7, 3.7, and 4.2 across all 4 chatbots, respectively, indicating that the criteria were at least either partially or completely met. All 4 chatbots received the lowest possible score of 1 on question 8, indicating inadequate description of the risks of each treatment. Additionally, all 4 chatbots received a score of 3 on question 9, indicating that their descriptions of what would happen if no treatment were used partially met criteria. Variations in DISCERN scores were noted between the chatbots with Microsoft CoPilot having the highest scores across all 3 sections, while Meta AI had the lowest.

Quality results of patient education materials from AI chatbots in other studies have been mixed. The previously mentioned study assessing the quality of information related to benign prostatic hyperplasia surgery from 4 chatbots (ChatGPT, Bard, Bing AI, and Doximity GPT) yielded a mean DISCERN score of 3.3.^[23]^ Another previously mentioned study assessing 100 cancer-related queries posed to 4 AI chatbots (ChatGPT, Perplexity, Chatsonic, and BingAI) found a median overall DISCERN score of 5, as well as median PEMAT scores of 66.7% for Understandability and 20.0% for Actionability, with Bing AI having the highest scores across both tools.^[21]^ Additionally, a study assessing the quality of patient education materials on cardiac catheterization produced by the same 4 chatbots that we analyzed in this study (ChatGPT, Microsoft Copilot, Google Gemini, and Meta AI) found that ChatGPT produced the highest quality material with an overall mean DISCERN score of 4.5 and PEMAT Scores for Understandability and Actionability of 100% and 75%.^[28]^ The variation in results may be due to the fact that we analyzed a different set of chatbots, or that the relative quality of material produced by AI chatbots may be improving with time. However, a constant seen across studies is the PEMAT Actionability scores are consistently lower than Understandability scores, which may be because chatbots have built-in limitations and always recommend consulting with your healthcare provider in their responses.

Studies have shown that the quality of patient education materials can be affected by the prompts entered into the AI chatbots. A study comparing ChatGPT and Google Search responses to questions about specific medical conditions found differing results when the questions sought general medical knowledge versus medical recommendations, with ChatGPT performing better than Google Search for general medical knowledge and Google Search performing better when providing medical recommendations.^[29]^ Another study assessing the quality and readability of information from ChatGPT on otolaryngology surgical procedures found that, if prompted to, ChatGPT could simplify the information and improve its readability. However, they also noticed that simplifying the information reduced its quality, with lower DISCERN scores after simplification.^[30]^ Also, while our investigation does not directly compare chatbot produced material to web-based material, it is important to note that previous literature has found variability when comparing PEMAT scores from both resources, with chatbots in some instances outperforming medical association webpages and vice versa.^[31,32]^ This flexibility and adaptability provided by AI chatbots represents a significant strength and highlights their future potential.

For ease of reference, Table 8 has been included depicting results of similar studies referenced in the discussion.

**Table 8:** Outcomes of similar studies referenced in Discussion section.

	Current Study	AI material related to 100 cancer related queries^[24]^	AI material related to Benign Prostatic Hyperplasia^[23]^	Readability of AI material related to cardiac catheterization^[3]^	Quality of AI material related to cardiac catheterization^[32]^	Assessment of web-page material related to Nasopharyngeal carcinoma^[25]^	Assessment of web-page materials related to esophageal cancer^[26]^
Mean Readability (FRES)	20.3	N/A	N/A	40.2	N/A	53.2	48.25
Mean Readability (Grade Level)	13.7	12.7	12.1	12.45	N/A	14.3	N/A
Median and Range of PEMAT Understan-dability scores	91.7% (median) 90.9–100% (range)	N/A	66.7% (median) 33–90% (range)	N/A	96% (median) 82%–100% (range)	N/A	N/A
Range of PEMAT Actionability scores	75% (median) 50–75% (range)	N/A	20% (median) 0–40%(range)	N/A	75% (median) 50–75% (range	N/A	N/A
Mean DISCERN	4.2	3.3	5 (median) 2–5 (range)	N/A	4.5	N/A	N/A

Limitations of this study include that we used chatbot responses from one search at a single point in time, so our findings are only applicable to those specific results. Given the ever-changing nature of chatbots, the generalizability of our findings is thereby limited. This may have an effect on which chatbot we concluded to have performed the best as AI chatbots are constantly changing and improving. Performing multiple searches, especially over different periods of time, may have been able to mitigate this. Another limitation is use of the PEMAT and DISCERN tools to assess quality, as they are validated only for evaluating patient education materials and written healthcare information on treatment choices, respectively. However, they have not been validated for material produced by AI chatbots, representing a significant limitation since we used them to have an objective way to assess quality. This fact may have affected which materials were deemed to be of higher quality. This highlights the need for a validated tool to assess materials generated by AI chatbots. Furthermore, PEMAT and DISCERN do not assess accuracy of patient education materials, which is an important consideration when seeking to evaluate their quality. Another limitation was the use of a single source, the Cleveland Clinic, to develop the questions we posed to the chatbots. Although likely having no effect on our results, using a broader set of questions may have been a more accurate representation of the true patient experience. This could have been mitigated by adding questions from websites of other well-known clinics. Finally, only 2 authors performed the quality assessments and disagreements were solved by discussion, which yields the possibility of bias.

## 5. Conclusion

AI chatbots, which contain patient education material at a reading level exceeding that recommended by the NIH and AMA, continue to produce high quality with readability and quality measures similar to that of web-page based material and variations in the quality and readability of materials between chatbots. Google Gemini provided the most promising readable and high-quality patient education materials on chemotherapy cardiotoxicity. Strengths of our study include the combined analysis of readability and quality which provide a more comprehensive assessment of chatbot generated material at a given point in time. Many similar papers typically analyze one or the other, however both are relevant to patient education. Another strength if the novelty of our area of investigation. To our knowledge, this is the first study assessing the quality and readability of patient education materials on chemotherapy-induced cardiotoxicity produced by AI chatbots. We recommend cautioning any patients who wish to use chatbots to generate patient education material about its readability concerns, but do not discourage their use as an educational tool. We recommend posing open ended questions when querying chatbots and always consulting with a physician before making healthcare decisions. Future studies are needed to continue assessing the effectiveness of AI chatbots in providing health information to patients, particularly if they can be prompted in a standardized way to improve the readability and/or quality of the material they generate. Future studies are also needed to assess how the readability and quality of chatbots are changing over time.

## Author contributions

**Conceptualization:** Benjamin J. Behers.

**Data curation:** Christoph Stephenson-Moe.

**Investigation:** Benjamin J. Behers, Rebecca M. Gibons, Brett M. Behers, Laura De Jesus Herrera, Djhemson Anneaud, Manuel A. Rosario, Caroline N. Wojtas, Samantha Bhambrah, Karen M. Hamad.

**Methodology:** Benjamin J. Behers.

**Writing – original draft:** Christoph Stephenson-Moe, Rebecca M. Gibons.

**Writing – review & editing:** Christoph Stephenson-Moe, Rebecca M. Gibons, Brett M. Behers, Laura De Jesus Herrera, Djhemson Anneaud, Manuel A. Rosario, Caroline N. Wojtas, Samantha Bhambrah, Karen M. Hamad.
